# Building Trust and Trustworthiness in Public Institutions: Essential Elements in Placing Trust at the Heart of Health Policy and Systems: Comment on "Placing Trust at the Heart of Health Policy and Systems"

**DOI:** 10.34172/ijhpm.8782

**Published:** 2024-10-02

**Authors:** David H. Peters

**Affiliations:** Faculty of Health, York University, Toronto, ON, Canada

**Keywords:** Trust, Trustworthiness, Health Systems, Social Cohesion, Public Institutions

## Abstract

In this commentary, I argue that societies are facing major crises in trust that extends well beyond health systems, outlining actions that can enhance trust in public institutions and benefit health systems. There are also areas where strengthening health systems can serve to build broader trust and social cohesion, such as by providing social protection and health services that are responsive to people’s needs. Understanding the dimensions of "trustworthiness" for different actors in a health system also provide insights on how to build, restore, and maintain trust. Whereas research evidence claims a foundational role for trustworthy intervention among health professions, other factors may be more influential for others. These include the credibility of the source, participation in the intervention with observably fair distribution of the benefits, the ethical behavior of key actors, reliability in service delivery and its results, transparent and consistent communications, and addressing breaches in trust.

 Health systems are inherently complex, relying on interactions between many key actors, including patients, their families, healthcare providers, policy-makers, funders, business, and the general public. Health systems also depend on trusting relationships between these actors to work effectively. In their editorial on *Placing Trust at the Heart of Health Policy and Systems*, McKee and colleagues succinctly describe many reasons why building and maintaining trust in health systems needs to be prioritized, arguing that it rarely is and why this must change.^1^ They make an appeal for better measurement of trust, in part by synthesizing ideas from various disciplines that have typically examined trust in isolation of each other. They also highlight the critical role played by key actors in demonstrating trustworthiness if trust in a health system is to be built, restored, and protected.

 I fully agree with their perspectives concerning health systems, and their assertion that trust in health systems reflect wider issues of trust in society. I would argue that many societies are facing larger crises in trust that threaten social cohesion and public institutions more broadly, and that the societal drivers for mistrust need to be understood and tackled to better address trust issues that are specific to health systems. And while assessing trust by stretching across disciplinary silos is needed, a focus on the multiple facets of “trustworthiness” merits deeper probing. In this commentary, I explore the following main questions:

1. How would a better understanding of the drivers of societal distrust help address trust issues specific to health systems?
2. How do characteristics of trustworthiness help to explain the phenomena of trust (and distrust) in health systems and other public institutions, and the ways those insights can be used to shape interventions?

The concepts of trust, trustworthiness, and distrust are massive and the subject of much debate across disciplines.^2,3^ Trust is a relational concept that involves a willingness to be vulnerable to another party based on positive expectations of their intentions or actions.^3^ Trustworthiness refers to the qualities or characteristics that make a party worthy of trust, such as perceived benevolence, integrity, competence, and predictability.^2,3^ Distrust is the absence of trust and involves negative expectations about another party’s intentions or behavior, often arising from past experiences of betrayal or disappointment (noting that the term “mistrust” is a similarly used, often in a more temporary or situational context).^4^

## Societal Distrust and Health Systems

 The decline in trust in public institutions is a global phenomenon, covering many areas of public life. These are related to institutional trust – which is associated with formal governmental and other public bodies, including health care systems, but also the media and other regulated industries (eg, banks) that typically have some degree of representative or delegated authority; as well as civic trust – which is related to belief in the authority and competence of governing institutions to act in ways are transparent and in the best interests of the common good; and social trust – which is often based on the belief that others are good neighbors and members of a community, which may be manifest through voluntary and often informal initiatives to address specific challenges in a particular locality, such as interfaith networks or neighbourhood crime watches. At the 2023 Summit for Democracy, United Nations Secretary-General António Guterres focused on democratic institutions, asserting that “The foundations of social cohesion and trust in democratic institutions are being rocked to the core.”^5^ His statement reflects concern over the growing skepticism towards institutions’ intentions, competency, and integrity which has fuelled the rise of populist movements across the world.

 Numerous surveys and reports have signalled growing distrust in public institutions around the world. In their international surveys, the Pew Research Center has found that while democracy is broadly popular, enthusiasm for democratic governance is often lukewarm.^6^ Many people feel that politicians and business leaders benefit more from democratic systems and market economies than ordinary citizens. This perception has contributed to the rise of populist and autocratic sentiments, with significant portions of the population in various countries expressing support for nondemocratic alternatives such as military rule.^6^ According to the 2023 Global State of Democracy report, many countries have seen notable declines in areas such as credible elections, effective parliaments, and judicial independence.^7^ This trend is evident in African countries, where coups and prolonged authoritarian rule have significantly undermined democratic institutions. In Asia, issues like corruption and the misuse of laws intended to address misinformation have eroded public trust, despite ongoing pro-democratic movements.^7^

 The correlation between distrust in democratic institutions and trust in health systems is also evident across multiple studies and surveys.^6,8,9^ Countries with higher trust in democratic institutions typically have stronger and more trusted health systems, while those with lower trust in government struggle with public health challenges. The cause-and-effect relationships are difficult to untangle, but the fact that these occur in many contexts and points in history suggests an interdependence.

 In many settings, it will be insufficient to simply focus on building trust within health systems alone if they are to be sustained. It is important to ensure that major public institutions are robust, transparent, accountable, and participatory to ensure effective public health governance. Our own experience showed that helping the Afghanistan government and non-governmental organizations to deliver effective, equitable, and trusted health services during the previous period of civilian rule, in part through use of transparent performance scorecards across the country,^10^ could not prevent nor withstand the larger societal pressures that accompanied the violent return of the Taliban regime in 2021. Tragically, thousands of health workers lives have been put at risk because of their dedication to the women’s health, or because their work had been supported by foreign governments and organizations. There are many cases that have shown how a lack of trust in government institutions can impede public health efforts and exacerbate health inequities, particularly during crises like the COVID-19 pandemic.^8,11^ It is likely that addressing underlying issues of trust in government and public institutions will remain instrumental in improving public health outcomes.

 The Open Government Partnership is an international initiative that aims to promote transparent, participatory, and accountable governance by fostering collaboration between governments and civil society, enhance the quality of governance and improve public trust in government institutions.^12^ The Open Government Partnership supports processes that have involved over 75 governments and thousands of civil society organizations around the world to make commitments to actions and accountability in their work across a range of areas, which I have categorized in Table 1. The first set of actions are typically led in sectors beyond health, it should be apparent that such actions can provide a foundation that reinforces trust-building in health systems. There are also good examples of participatory governance in the health sector. One example is the CONNECT Initiative, where communities in Laos have been empowered to participate in health decision-making processes, emphasizing governance transparency and accountability, and leading to more responsive and effective health services.^13^ The bottom section of the table highlights some of the most direct areas of interdependency with the health system, involving actions that foster economic inclusivity and social protection, or improve government responsiveness and service delivery, where health systems reforms may play leading roles.

**Table 1:** Key Actions for Building Trust in Public Institutions Beyond Health Systems

**Key Action**	**Description**	**Example**
**Areas Where Leading Roles are Typically Dependent on Actions Outside the Health Sector**		
A. Strengthen rule of law and justice	Ensure laws are applied fairly and consistently, with independent and transparent judicial processes.	*Independent anti-corruption bodies* and a judiciary free from political influence.
B. Enhance civic participation and engagement	Involve citizens in governance through civic education and public consultation.	*Participatory budgeting* processes that allow citizens to influence public spending decisions.
C. Promote government transparency	Increase the openness of government operations, making information and data accessible to the public.	*Freedom of Information laws* that allow citizens to request and obtain government records.
D. Demonstrate ethical leadership and integrity	Ensure public officials adhere to high ethical standards and commit to public service.	*Conflict of interest policies* and regular ethical training for public officials.
**Areas Where Health Systems Play Can Play a Leading Role**		
A. Foster economic inclusivity and social protection	Implement policies to reduce economic inequality and provide social safety nets.	*Social welfare programs* like unemployment benefits and *universal healthcare* to reduce poverty and inequality.
B. Improve government responsiveness and service delivery	Increase the efficiency and effectiveness of government services to meet public needs.	*One-stop government service centers* that streamline processes and reduce bureaucracy. *Access to person- centred healthcare* that improves health and wellness.

## Trustworthiness and Evidence

 McKee and colleagues convincingly describe trust as a multi-faceted construct that is highly context specific, and changeable over time. These features make trust difficult to measure, but also raise larger concerns about the trustworthiness of the evidence around the relationships between interventions (eg, policies, programs, clinical or behavioral actions) and trust. It is an almost tautological assumption that trustworthiness about how well an intervention works (ie, validity of the evidence) will influence people’s trust in an intervention and the individuals or organizations that deliver them. Trustworthiness among health professionals is heavily influenced by the traditions of evidence-based medicine and public health,^14,15^ and more recently by the evidence-based management movement.^16^ Research evidence plays a primary role among researchers and health professionals as the basis for trustworthiness, with the level of trustworthiness dependent on study design, the quality of data collection and analysis methods, and the strength of the findings. But often policy-makers and the general public are poorly equipped to directly use research evidence, and rely on other sources of evidence. These include tacit knowledge and judgement of influential (ie, “trusted”) organizations, compelling stories, opinions of those who share their values and priorities, as well as interpretations based on their own experience. Among the general public, and particularly among social media users, the explosive growth of social media has fostered a wide range of “influencers” who are considered credible sources of information on health care and wellness that is well beyond their expertise. They may be seen as more authentic and relatable than experts or other types of celebrities. This creates parasocial relationships that are one-sided yet allows followers to feel a personal connection to them regardless of their qualifications. They can effectively use emotional appeals that are amplified though algorithms that prioritize engaging content and popularity rather than credibility.

 Social media also has also become a significant factor in affecting trust in broader democratic institutions around the world. One study on the role of social media on politics and society in 19 countries showed wide variation, with most countries perceiving social media as good for democracy in the vast majority of countries (eg, by raising public awareness, drawing the attention of policy-makers to critical issues, and helping people to be more accepting of people from different ethnic groups, religions and races), but with mostly negative effects in the United States.^17^

 These multiple perspectives of trustworthiness support the assertions of McKee and colleagues – trust should be measured repeatedly by different methods and lenses of analysis and interpretation. Its multi-faceted nature also has implications for how interventions can be designed to build trust, or how those interventions that depend on trust can be successfully implemented.

Table 2 summarizes some of the main grounds for belief in the trustworthiness in health systems and other public institutions and provides insights on ways to influence trust. Some characteristics, such as research evidence, are focused on interventions and their effects, and has typically been the domain of scientists and experts. Other factors focus on the credibility of the source of the information or how an intervention is delivered, based on the criteria that supports their credibility (eg, competence, benevolence, ethical behaviour, or their popularity and authenticity). People’s participation in the design and implementation of policies, and the observation of fair distribution of benefits are other important considerations for building trust. Cutting across each of these considerations is the importance of transparent and consistent communication, and responses to breaches of trust.

**Table 2:** Key Features of Trustworthiness in Public Institutions and Health Systems

**Trustworthiness Factor**	**Description**
A. Robust research methodology	Rigorous, transparent, and reproducible methodologies are intended to ensure that research and evidence are valid and can be trusted.
B. Credibility of source	Perceived competence and benevolence of the source (individuals or institutions) contribute to the credibility of the information or intervention provided. High visibility, authentic storytelling, audience engagement and emotional appeal can also instill trustworthiness in the absence of scientifically sound advice.
C. Participation in design, implementation, and benefit	Involving implementers and intended beneficiaries in the design and implementation of policies, with observably fair distribution of the benefits, helps to ensure that institutions are relevant and trusted.
C. Reliability of implementation and results	Reliable implementation and consistent results reinforce trust by demonstrating that policies work as intended.
D. Ethical behavior of key actors	Integrity of action, attention to equity, and focus on marginalized groups are critical for ethical behavior and the public trust.
E. Consistency and transparency in communication	Consistent and transparent communication builds trust by keeping the public informed and involved, as well as those charged with implementation.
F. Addressing causes of mistrust	Proactively addressing causes of mistrust, especially when trust has been breached, is essential for rebuilding trust.

 By focusing on these strategies to improve trust in public institutions, and paying attention to trustworthiness in health systems, all actors—policy-makers, practitioners, researchers, governments, businesses, non-governmental organizations, the media and communities themselves, including the most marginalized members—can help to assure that both public institutions and health systems are trusted and effective, ultimately improving public health and societal cohesion.

## Ethical issues

 Not applicable.

## Conflicts of interest

 Author declares that he has no conflicts of interest.

## References

[1] McKee M, van Schalkwyk MC, Greenley R, Permanand G (2024). Placing trust at the heart of health policy and systems. Int J Health Policy Manag.

[2] Harrison McKnight D, Chervany NL. Trust and distrust definitions: one bite at a time. In: Falcone R, Singh M, Tan YH, eds. Trust in Cyber-Societies. Berlin: Springer; 2001:27-54. 10.1007/3-540-45547-7_3.

[3] Colquitt JA, Scott BA, LePine JA (2007). Trust, trustworthiness, and trust propensity: a meta-analytic test of their unique relationships with risk taking and job performance. J Appl Psychol.

[4] Kramer RM (1999). Trust and distrust in organizations: emerging perspectives, enduring questions. Annu Rev Psychol.

[5] United Nations Secretary General. Press Release. Strong Democratic Societies Can Enable Non-Violent Change, Secretary-General Tells Summit, Calling for New Social Contract between Governments, Their People. UN Press; 2023. https://press.un.org/en/2023/sgsm21752.doc.htm. Accessed August 26, 2024.

[6] Wike R, Fetterol J. Global Public Opinion in an Era of Democratic Anxiety. The Pew Charitable Trusts; 2022. https://www.pewtrusts.org/en/trust/archive/spring-2022/global-public-opinion-in-an-era-of-democratic-anxiety.

[7] International Institute for Democracy and Electoral Assistance. The Global State of Democracy 2019: Addressing the Ills, Reviving the Promise. https://www.idea.int/publications/catalogue/summary-global-state-of-democracy-2019?lang=en.

[8] Edelman. 2023 Edelman Trust Barometer: Navigating a Polarized World. https://www.edelman.com/trust/2023/trust-barometer.

[9] Wellcome Global Monitor 2020: COVID-19. How COVID-19 Affected People’s Lives and Their Views About Science. https://wellcome.org/reports/wellcome-global-monitor-covid-19/2020.

[10] Edward A, Kumar B, Kakar F, Salehi AS, Burnham G, Peters DH (2011). Configuring balanced scorecards for measuring health system performance: evidence from 5 years’ evaluation in Afghanistan. PLoS Med.

[11] Edelman. 2022 Edelman Trust Barometer Special Report: Trust and Health. https://www.edelman.com/trust/22/special-report-trust-in-health.

[12] Open Government Partnership Secretariat. Open Government Partnership Global Report: Democracy Beyond the Ballot Box. 2019. https://www.opengovpartnership.org/campaigns/global-report/#downloads.

[13] Haenssgen MJ, Elliott EM, Phommachanh S, Souksavanh O, Okabayashi H, Kubota S (2024). Community engagement for stakeholder and community trust in healthcare: short-term evaluation findings from a nationwide initiative in Lao PDR. Soc Sci Med.

[14] Sackett DL (1997). Evidence-based medicine. Semin Perinatol.

[15] Brownson RC, Baker EA, Leat TL, et al. Evidence-Based Public Health. Oxford University Press; 2003.

[16] Barends E, Rousseau DM. Evidence-Based Management: How to Use Evidence to Make Better Organizational Decisions. New York: Kogan Page Publishers; 2018.

[17] Pew Research Center. Social Media Seen as Mostly Good for Democracy Across Many Nations, But U.S. is a Major Outlier. 2022. https://www.pewresearch.org/global/2022/12/06/social-media-seen-as-mostly-good-for-democracy-across-many-nations-but-u-s-is-a-major-outlier/.

